# Postpartum Pelvic Instability: A Case Report

**DOI:** 10.7759/cureus.33707

**Published:** 2023-01-12

**Authors:** David Sosnoski

**Affiliations:** 1 Orthopedic Surgery, Beaumont Health, Farmington Hills, USA

**Keywords:** pregnancy, postpartum, pelvis, pelvic diastasis, pelvic instability, pelvis surgery

## Abstract

Pubic symphysis diastasis following childbirth is a rare orthopedic condition that can be debilitating in the postpartum period. There have been treatment options documented, ranging from conservative to surgical; however, no standard of care has been established. We present a 44-year-old female patient who underwent open reduction and internal fixation for continued instability from postpartum pubic symphysis diastasis with a good overall outcome. We demonstrate good outcomes in a patient treated with surgical fixation of postpartum pelvic diastasis. We hope to deliver insight to future orthopedic surgeons with the challenges in treating this condition.

## Introduction

Pubic symphysis diastasis following childbirth via vaginal delivery is considered a rare but debilitating condition. It is widely known that the pubic symphysis joint is cartilaginous in nature, and widening of this joint in pregnancy in childbirth is a physiologic change to allow adequate expansion for successful labor and delivery [1]. However, there has been documented literature of pubic diastasis considered to be nonphysiologic, defined as greater than 1 centimeter, that can leave new-found mothers in persistent pain with residual debility. The incidence of pubic symphysis diastasis is reported to be 1 in 300 to 1 in 30,000 childbirths, with many likely misdiagnosed or not diagnosed altogether [1,2]. These injuries pose a challenge in decision-making for the treating orthopedic surgeon, as these women have heightened risk for surgery in the peripartum state, partnered with ongoing debility that can affect the care of their newborn. We present the following intriguing case of a 44-year-old female who sustained an atraumatic pelvic ring injury with complete pubic symphysis diastasis of 1.2 centimeters successfully treated with open reduction and internal fixation after chronic instability and persistent debility with failed conservative management.

## Case presentation

Our patient is a 44-year-old female who initially presented to our institution to give birth to her sixth child. She presented at 40 weeks four days gestation for scheduled induction of labor for advanced maternal age. Active labor was initiated with Pitocin augmentation and epidural spinal anesthesia. Per medical records, the patient delivered a baby boy at 9lb 2 oz (4139 grams) approximately four hours following induction. The patient had a minor perineal laceration, which was closed primarily with sutures. Otherwise, no birthing complications were noted. The patient then underwent mini-laparotomy tubal ligation the following morning without complication.

**Figure 1:**
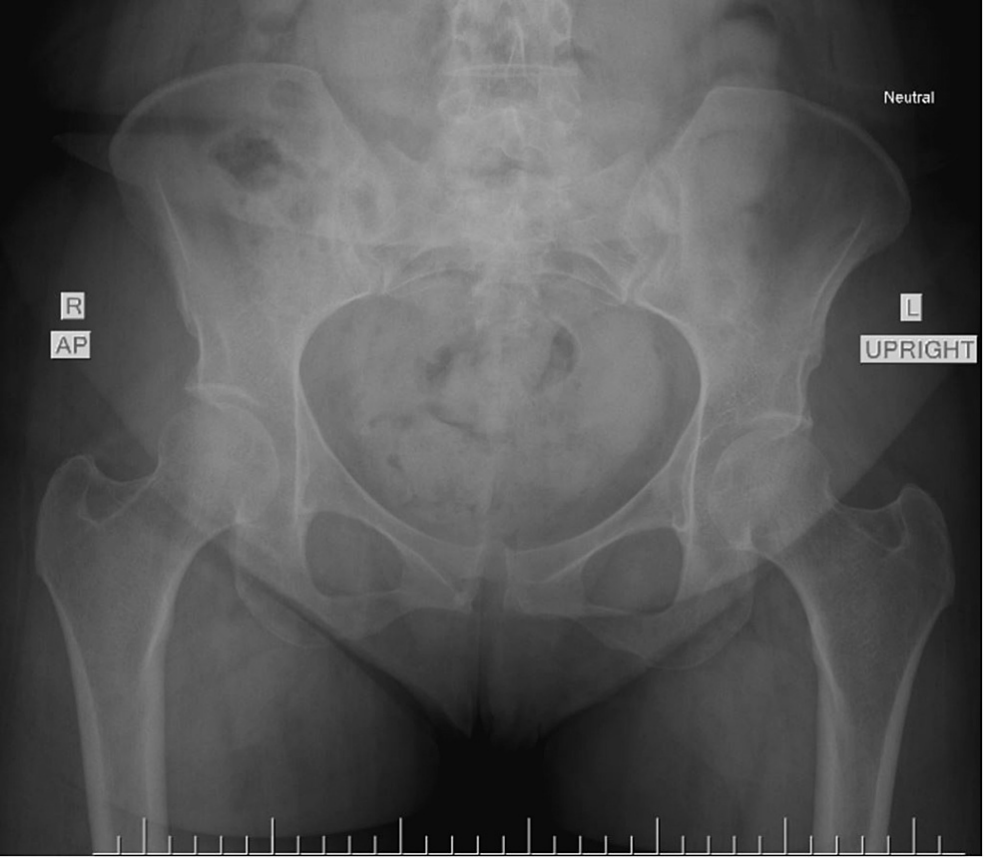
Neutral anteroposterior (AP) radiographic view of the pelvis in our patient

The patient reported right lower extremity pain and swelling with ambulation in the days following her delivery, necessitating a walker for assistance due to reported imbalance. Duplex venous ultrasound was found to be negative for deep venous thromboembolism (DVT). A neutral anteroposterior radiograph was taken at the time shown in Figure 1.

She was subsequently discharged with home physical therapy daily; however, she experienced continued debility, which progressed to wheelchair dependency noted at her six-week postpartum obstetrician follow-up appointment. She subsequently saw a physiatrist with an MRI lumbar spine ordered, demonstrating no acute pathology, and prescribed continued physical therapy. She was then evaluated at her eight-week postpartum visit with an anteroposterior (AP) radiograph of the pelvis, revealing diastasis of the symphysis measuring 1.2 centimeters and suggestive widening of her posterior sacroiliac joints.

**Figure 2:**
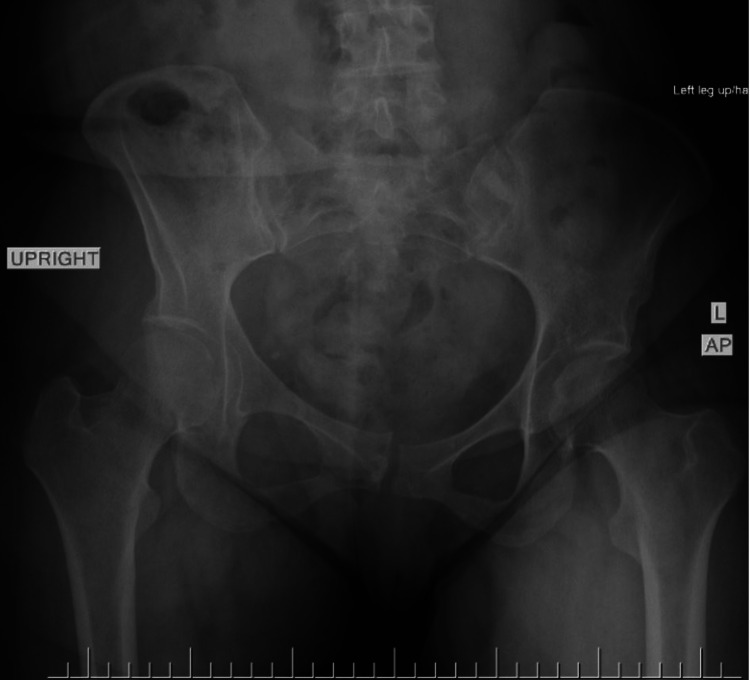
Anteroposterior (AP) radiographic view of the pelvis as a stress view, with the left leg elevated, demonstrating incongruity of the pubic symphysis along with diastasis, defined as pelvic ring instability

**Figure 3:**
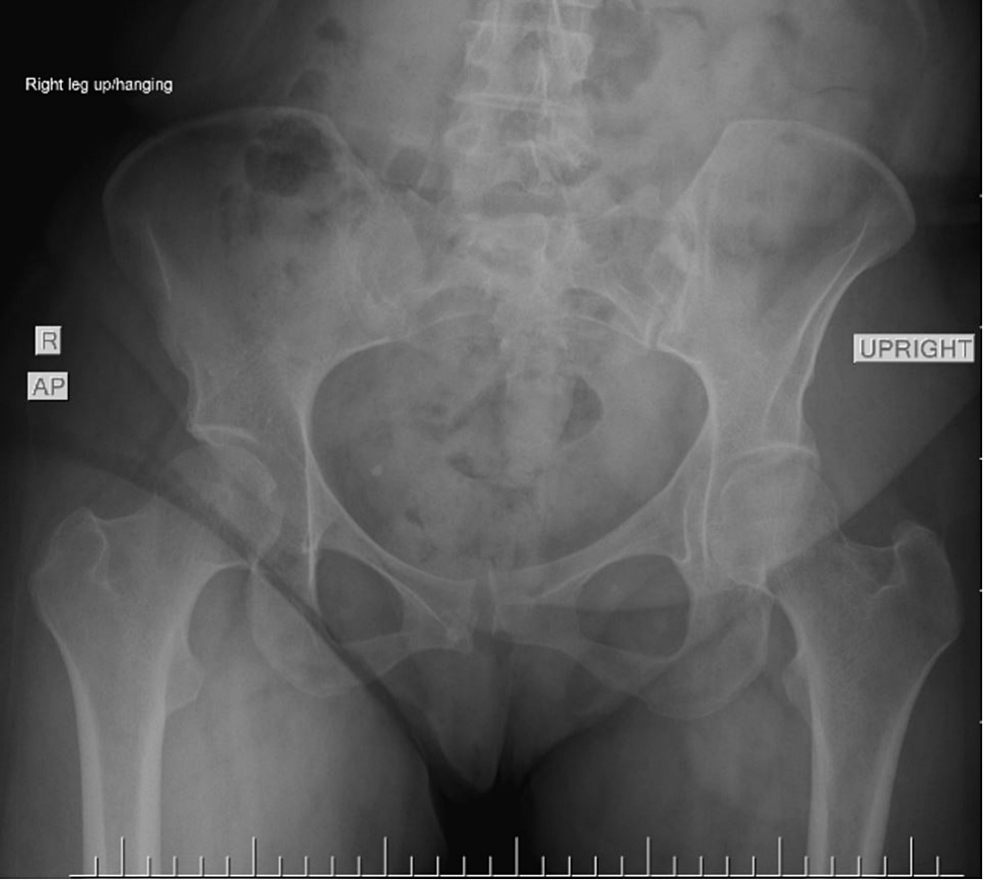
Anteroposterior (AP) radiographic view of the pelvis as a stress view, with the right leg elevated, demonstrating pubic symphyseal widening along with widening of the patient's left sacroiliac (SI) joint, defined as pelvic ring instability

**Figure 4:**
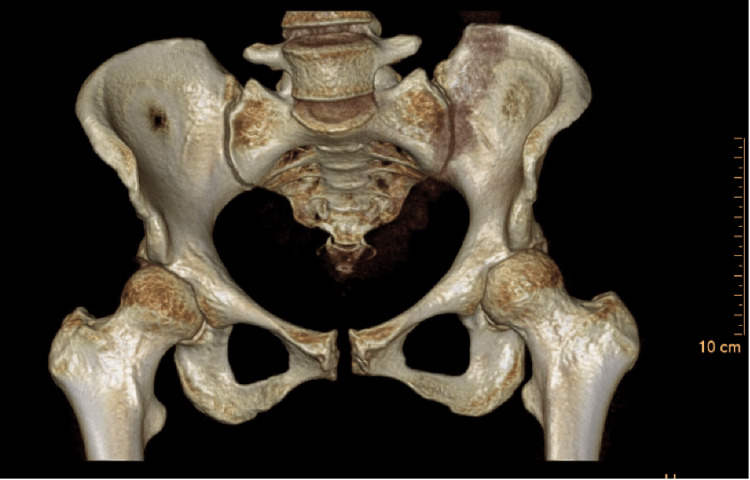
Computed tomography (CT) scan 3D reconstruction of the patient's pelvis demonstrates pubic symphyseal widening

**Figure 5:**
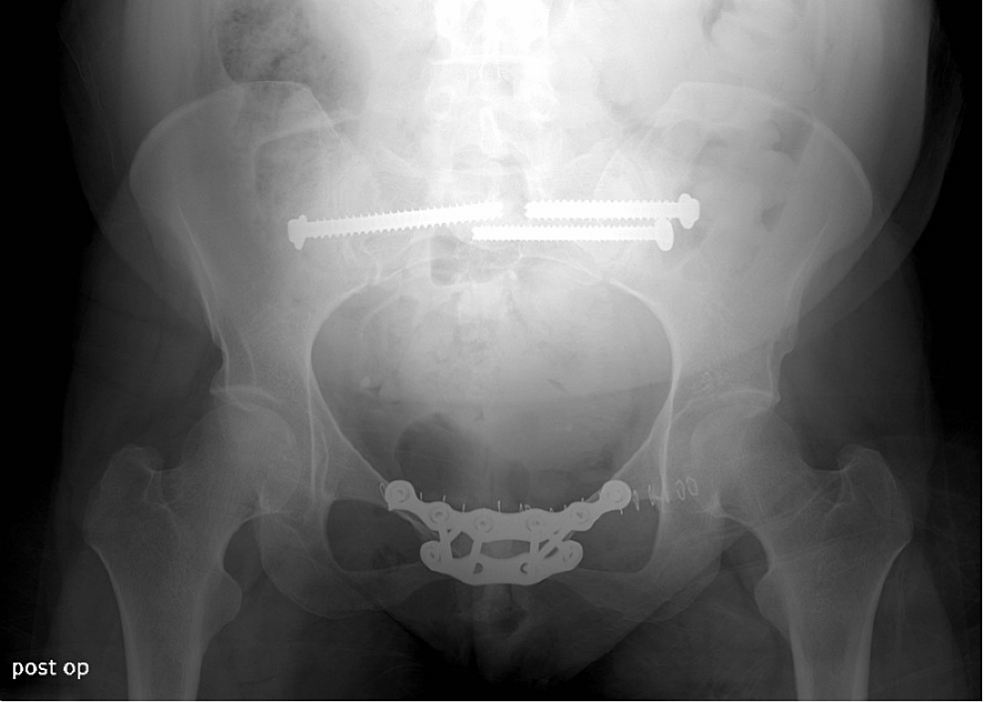
Anteroposterior (AP) radiographic view of the patient's pelvis taken postoperatively, demonstrating the orthopedic hardware in position

This was again demonstrated on a repeat AP radiograph of the pelvis four months postpartum. The patient was referred to our orthopedics team after five months of conservative management without improvement. Stress views of her pelvis in the orthopedic clinic (Figures 2, 3) confirmed symphysis instability with left sacroiliac joint widening. At an orthopedic visit six months postpartum, a long discussion of treatment options was had, including continued nonoperative versus operative. With the lack of improvement with exhausting conservative measures and the patient still with the inability to weight-bear without pain or feelings of instability, the patient elected to proceed with operative intervention at that point in time. Computed tomography (CT) scan of the pelvis was ordered for surgical planning, as seen in Figure 4. The patient was then treated with open reduction and internal fixation with two pubic symphysis anterior plates and screws along with in-situ bilateral sacroiliac screw fixation without complications (Figure 5).

The patient was made weight-bearing as tolerated to bilateral lower extremities for transfers only and necessary household ambulation with a walker following the procedure for eight weeks, along with physiatry-led rehabilitation. At her two-week, one-month, and three-month follow-up appointments, she continued to experience postoperative pain and difficulty with ambulation. At her visit four months postoperatively, she demonstrated improvements in pain and strength with a full range of motion, ambulating with a cane. At her six and 12-month follow-up visits, she walked without sn assistive device and without pain. Follow-up radiographs demonstrated continued stability and maintenance of alignment of orthopedic hardware.

## Discussion

Postpartum pubic symphysis diastasis (PSD) following normal spontaneous vaginal delivery has been documented as a rare condition. As previously noted, the incidence of peripartum pubic separation varies from 1 in every 300 to 1 in 30,000 deliveries, with many going undiagnosed [1,2]. Differential diagnoses for PSD include sciatica, lumbar radiculopathy, osteitis pubis, and osteomyelitis which can make diagnosis difficult [3]. A common complaint of women during and following pregnancy includes both groin and pubic pain, which is likely due to the physiologic separation of the pubic symphysis. There is ligamentous relaxation at the pubic symphyseal joint along with widening of the sacroiliac joint due to actions by relaxin and progesterone, enabling normal vaginal delivery [2]. However, an increase in this separation due to increased stress during delivery may result in PSD. PSD is defined as a pathologic separation of the pubic symphysis of more than 10 mm [4]. It has also been documented that diastasis of more than 14 mm is usually associated with damage to the sacroiliac joint and potential disruption of one or both sides of the sacroiliac ligaments [5,6]. Risk factors that have been identified for postpartum PSD include women with their first pregnancy, prolonged active labor, multiple gestations, and macrosomia [2]. Normal physiologic changes of pregnancy should be considered when exploring and selecting treatment options for this troublesome injury. Pregnancy, along with bed rest during the peripartum period, are both associated with an increased risk of deep venous thrombosis, which can inherently be increased with possible surgical intervention [2].

The treatment modality deemed most suitable for postpartum pubic symphysis diastasis is still currently heavily debated. Conservative treatment has been described as the treatment of choice in pelvic symphysis diastasis [3]. There are multiple documented case reports describing success with nonoperative management, including the use of pelvic binders, temporary immobilization, bed rest, and pain medication regimens [3,7-9]. A more recent report also vetted weight-bearing to tolerance and the use of intense physical therapy along with pelvic floor exercises as successful conservative treatment measures [10]. All of the reports mentioned above claim excellent results with symptomatic improvement at the end of follow-up, with initial diastasis measuring from 2.3 cm to 7.3 cm.

Yoo et al. also reported that of the 11 patients followed, nine were successfully treated nonoperatively. They also describe that five of the 11 patients had persistent dysfunction and remained symptomatic at the last reported follow-up [2]. Other previous cases have also described suboptimal results with continued pain and debility [11,12]. And although specific recurrences are difficult to predict, it has been documented that there is a high recurrence rate of 68-85% in future pregnancies [3].

Patients with continued diastasis resulting in pain, instability, and debility in activities of daily living are offered other means of management once conservative measures have been exhausted. Operative management options to treat ongoing instability have been described as the placement of an external fixator, symphysiodesis, and anterior internal fixation of the symphysis with plates and screws, coupled with or without the use of sacroiliac screw fixation with minimal complication [4,12-15].

As an example of the above, Kharrazi et al. reported four patients with pubic symphysis widening averaging 6.4 cm measured postpartum, who were all initially treated nonoperatively with pelvic binder and bed rest [11]. Their report indicated although there were mild improvements if diastasis radiographically, all four continued to experience residual pain and disability related to their pelvic injury at their last follow-up. In conclusion, they advocated for formal examination under anesthesia and operative anterior plate fixation with unstable symphyseal diastasis, especially greater than 4 cm. 

Other cases of operative management, including open reduction internal fixation (ORIF) of the pubic symphysis widening, have also been described. Pubic symphysis arthrodesis with or without sacroiliac arthrodesis has been described historically with mixed results [13]. Early surgical intervention with anterior pubic symphysis fixation with plates and screws and adjunctive posterior stabilization as sacroiliac screws have been described in the past with success in patients who failed conservative management [4]. Najibi et al. also depicted ten patients treated operatively with ORIF of the diastasis. They report three patients with excellent results after treatment, four had good outcomes, and three patients left with fair to poor outcomes [12]. Lastly, Rommens et al. provided results of successful operative fixation in three patients that failed conservative management, with diastasis ranging from 1.5 cm to 4.5 cm [14]. Successful operative management has been described above overall; however, it has been noted that in patients that wish to continue to have children, that vaginal delivery is contraindicated following operative fixation due to the rigidity of the construct rendering the pelvis unable to widen as in normal physiologic vaginal delivery. 

While our patient initially presented with a diastasis of only 1.2 cm, conservative management had failed with physical therapy and protected weight bearing, as evidenced by the patient's continued pain with ambulation, instability, and continued disability caring for her newborn child. Placement of the anterior plate and screws coupled with three sacroiliac screws supplied excellent stability of the pelvic ring, as evidenced by repeat examinations during the patient's 12-month follow-up postoperatively. Although the patient was slow to progress initially, she demonstrated ambulation without assistance and the ability to care for her child without difficulty following her six-month postoperative visit.

## Conclusions

The expected prognosis is overall favorable for most individuals experiencing postpartum pubic symphysis diastasis (PSD). Most patients can expect a complete recovery without the persistence of pain and related symptoms. Radiographs obtained in follow-up office visits of patients who were treated conservatively can demonstrate improvement in the closure of the pubic symphysis; however, one cannot correlate the imaging findings to full symptomatic resolution in these debilitated patients. Operative management with internal fixation may be required to obtain clinical improvement and symptomatic relief following the failure of conservative management of greater than six months with continued instability of the pelvic ring. Some patients require a preoperative and postoperative period of physical therapy, including our patient presented above. No definitive recommendations exist regarding the management of pregnancies complicated by PSD, which would be a perceptive topic of research and discussion for a future prospective study. We hope this case study provides additional management insight not only to treating orthopedic surgeons but also to an entire multifaceted healthcare team providing paramount care to patients affected by PSD in the future. 
